# Projected climate change impact on oceanic acidification

**DOI:** 10.1186/1750-0680-1-2

**Published:** 2006-06-27

**Authors:** Ben I McNeil, Richard J Matear

**Affiliations:** 1 Climate & Environmental Dynamics Laboratory, School of Mathematics, University of New South Wales, Sydney, NSW, Australia; 2 CSIRO Marine Research and Antarctic, Climate and Ecosystem CRC, Hobart, Australia

## Abstract

**Background:**

Anthropogenic CO_2 _uptake by the ocean decreases the pH of seawater, leading to an 'acidification' which may have potential detrimental consequences on marine organisms [1]. Ocean warming or circulation alterations induced by climate change has the potential to slowdown the rate of acidification of ocean waters by decreasing the amount of CO_2 _uptake by the ocean [2]. However, a recent study showed that climate change affected the decrease in pH insignificantly [3]. Here, we examine the sensitivity of future oceanic acidification to climate change feedbacks within a coupled atmosphere-ocean model and find that ocean warming dominates the climate change feedbacks.

**Results:**

Our results show that the direct decrease in pH due to ocean warming is approximately equal to but opposite in magnitude to the indirect increase in pH associated with ocean warming (ie reduced DIC concentration of the upper ocean caused by lower solubility of CO_2_).

**Conclusion:**

As climate change feedbacks on pH approximately cancel, future oceanic acidification will closely follow future atmospheric CO_2 _concentrations. This suggests the only way to slowdown or mitigate the potential biological consequences of future ocean acidification is to significantly reduce fossil-fuel emissions of CO_2 _to the atmosphere.

## Background

Rising atmospheric CO_2 _concentrations via fossil fuel emissions will lead to an increase in oceanic CO_2 _via thermodynamic equilibration. Carbon chemistry in seawater undergoes the following equilibrium reactions as CO_2 _enters the ocean.

*CO*_2 _+*H*_2_*O *⇔ *H*_2_*CO*_3 _⇔  + *H*^+ ^⇔  + 2*H*^+ ^    (1)

The pH of seawater is defined by the amount of H+ ions available: *pH *= -log_10_[*H*^+^]. Increasing CO_2 _concentrations in the surface ocean via anthropogenic CO_2 _uptake will have implications for oceanic pH. As shown in equation (1), when CO_2 _dissolves in water it forms a weak acid (H_2_CO_3_), dissociates to bicarbonate generating hydrogen ions (H+), which makes the ocean less basic (pH decreases). Using an ocean-only model forced with atmospheric CO_2 _projections (IS92a), Caldeira and Wickett [4]predicted a pH drop of 0.4 units by the year 2100 and a further decline of 0.7 by the year 2300.

Future acidification (lowering of pH) may adversely impact marine biota, but our present understanding of the potential biological response is limited [1]. It is recognised however that a decrease in pH will alter the acid-base balance with the cells of marine organisms [1]. Marine organisms regulate intercellular pH by the metabolic interconversion of acids and bases, the passive chemical buffering of intra- and extra-cellular fluids, and the active ion transport (e.g. proton transport by extra-cellular respiratory proteins such as hemoglobin) [5]. Acid-base imbalances in marine organisms can lead to the dissolution of exoskeletal components such as calcareous shells, metabolic suppression, reduced protein synthesis and reduced activity [6,7]. Experiments to determine the likely response of marine organisms to pH changes have induced large changes in pH under laboratory conditions (>1) [8-13]. Little is known on what the gradual long-term effects of pH lowering will be on marine organisms. As pH changes have the potential to directly impact marine biota it is important to understand the magnitude of these changes under elevated CO_2 _levels and global warming.

Projections of future decreases in pH have been obtained from an ocean-only model that has not considered the effect of climate change feedbacks on the carbon chemistry of the ocean [4]. Recently, a study explored the role that climate change plays on the extent of ocean acidification [3]. Using three separate climate models they found climate change to insignificantly impact the projected future decreases of pH. However there was no investigation into this outcome even though the same models used predict large reductions in oceanic CO_2 _uptake due to climate change in association with temperature, circulation and biological feedbacks [2]. In this study we use a climate model to examine, partition and discuss the dominating climate change feedbacks controlling the future surface ocean pH.

## Results and discussion

**Figure 1:**
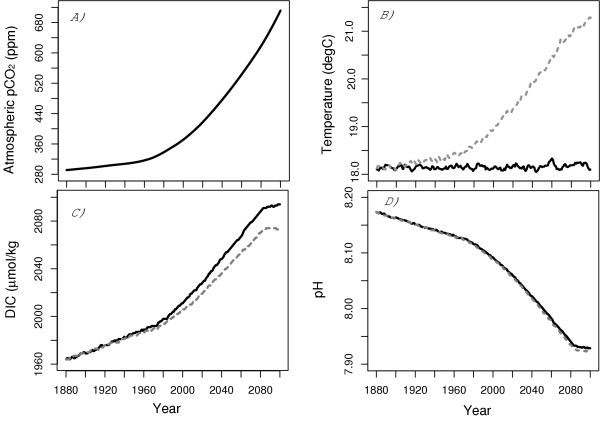
A) IS92a atmospheric CO_2 _projections used by our model; B) globally average sea surface temperature from the control experiment (solid line) and climate change experiment (dashed line); C) globally averaged Dissolved Inorganic Carbon (DIC) concentration (μmol/kg); D) globally averaged pH.

Changes in surface pH reflect changes in the speciation of carbon within the ocean and are a function of temperature, salinity, alkalinity and DIC concentrations. With climate change, the model projects an average surface temperature (SST) to warm from 18°C to about 21.5°C by the year 2100 (Figure 1b) while the globally averaged sea surface salinity (SSS) freshens from 34.71 to 34.53. The salinity normalized Alkalinity remained nearly constant at an average global concentration of 2270 μmol/kg. With climate change, we project by 2100 that the surface ocean DIC concentration is 18% less than the control experiment (reduction in DIC growth from 135 μmol/kg to 110 μmol/kg; see Figure 1c). The reduced growth in DIC concentration with climate change largely reflects reduced solubility of CO_2 _in the surface water due to the warming. We find pH decreases to be insensitive to climate change with virtually no difference between the transient and control experiment (Figure 1d). For both experiments, the globally averaged pH is projected to decrease from 8.17 in the year 1880 to about 7.91 by 2100.

**Figure 2:**
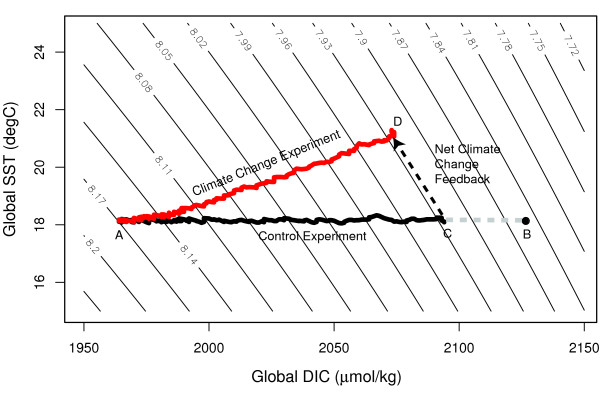
Evolution of mean surface pH in relation to DIC and sea surface temperature for both the control experiment (solid black line) and climate change experiment (solid red line). The *net *climate change feedback is shown as the dashed black vector between the control and climate change experiments. Point A is the initial state in the year 1880 before industrialization. Point B is the pH state (~7.82) in the year 2100 if the ocean absorbed atmospheric CO_2 _under equilibrium proportions. Point C is the pH state (~7.93) in the year 2100 for the control experiment and is equivalent to an oceanic steady state solution. Point D is the pH state (~7.93) for the year 2100 under climate change, and includes feedbacks such as circulation, biological production and temperature.

The insensitivity of pH to climate change is associated with compensating effects related to the ocean warming feedback. Figure 2 better illustrates the influence of DIC and sea surface temperature (SST) on pH in relation to the evolution of both the control and climate change experiments from the model. The evolution of pH from 1880 to 2100 for the control experiment is illustrated by line A-C, while line A-D in Figure 2 is the evolution of the climate change experiment. In the control experiment, there is no change in SST while oceanic uptake of anthropogenic CO_2 _increases DIC concentration (by ~135 μmol/kg), which consequently lowers pH considerably. Under climate change, SST increases while DIC concentration increases to a lesser extent than for the control (by ~110 μmol/kg). The difference between points C and D shows the net affect of climate change on pH. For pH, point C and D (net climate change feedback) lie almost exactly on contours of constant pH, therefore implying that climate change has no net affect on projected declining pH.

The solubility driven reductions in the growth of surface DIC concentration due to warming increase pH by a magnitude that is almost equal to pH decline directly associated with ocean warming, which cause the two affects to almost cancel each other. In Figure 2, the lines of constant pH are almost parallel to slope of the . As a consequence, the projected global pH decline of the climate change experiment does not differ from the projection made with the control experiment.

**Figure 3:**
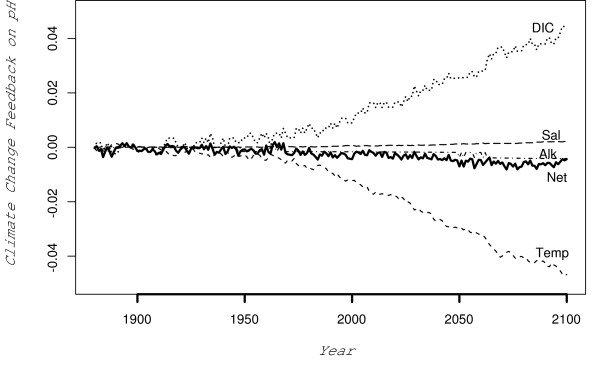
Net climate change effects on pH between 1880 and 2100 due to various controlling parameters. Negative pH change implies that climate change will amplify the reduction in pH from the control simulation, while a positive pH implies that climate change will buffer (or reduce) the decline in pH from the control simulation. Solid line represents the overall net climate change feedback while the dashed lines indicate changes due to DIC (which are solubility driven), direct effects of Temperature (Temp), Alkalinity (Alk) and Salinity (Sal).

To investigate the importance of different water properties changes on global-averaged pH, we compare the change in pH between the control experiment and climate change experiment for each individual water property change (ie. ). Future variations in salinity and alkalinity have little effect on pH, while the direct effects of ocean warming (SST) and indirect effects on DIC (solubility induced changes) dominate (Figure 3). For pH, the negative feedback associated with a reduction in growth of surface DIC concentrations due to solubility is offset by the positive feedback associated with the direct effects of ocean warming (Figure 3). The overall net climate change feedback impact on pH is small. However, as discussed earlier climate models show different sensitivities and it is unclear whether this result is unique to the CSIRO climate model. There is circumstantial evidence to suggest this phenomena may be independent of the type of climate model used. The IPSL climate model has a lower sensitivity (~3.6°C) but was found to undergo similar pH insensitivity as to the CSIRO climate model in Orr et al. (2005). Analysis on models with a broad range of sensitivities will further elucidate if our results are more indicative of climate models in general.

The CO_2 _biological pump within our simulations changed considerably with carbon export decreasing with climate change [2]. These changes would also lead to changes in pH within the water column however in the surface ocean, biologically mediated pH changes were found to be negligible.

**Figure 4:**
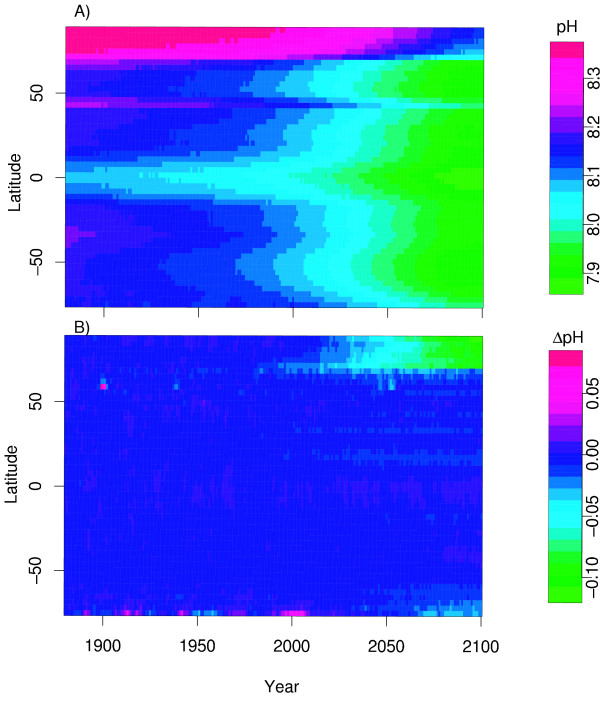
A) Zonally averaged temporal evolution of surface ocean pH from the climate change experiment up to the year 2100; B) Simulated zonally averaged evolution of net climate change feedback on surface ocean pH.

Figure 4a shows the zonal evolution of pH in the surface ocean up to the year 2100. Both the pH distribution along with it decline is zonally relatively uniform, decreasing from about 8.2 to 7.9 although the Arctic Ocean is more basic (~8.3). Figure 4b shows the zonal evoution of pH associated with the net climate change feedback. There is very little variation in the magnitude and structure of the meridional change in pH due to climate change. In the Arctic Ocean (>60°N) however, there is a positive feedback and a faint positive feedback in the high Southern Ocean (>65°S) beyond the year 2070. For these regions, climate change reduces sea-ice extent thereby allowing more absorption of anthropogenic CO_2 _independent of ocean warming which reduces pH beyond that of other parts of the ocean.

## Conclusion

Our study confirms previous suggestions that climate change feedbacks do not influence the projected decline in pH. This insensitivity to climate change occurs because the decrease in pH due to warming is nearly equal to but opposite in magnitude to the pH increase associated with reduced growth of DIC concentration in the upper ocean caused by reduced solubility of CO_2 _with ocean warming (Figure 2). Therefore, projections that neglect climate change [4] provide a reasonable estimate of the future pH change. Future projections of ocean acidification will therefore mainly be dependent on the future level of atmospheric CO_2_. The consequences of a small but sustained decrease in oceanic pH on marine phytoplankton are virtually unknown. It will be important for marine ecologists in the future to better understand the sensitivities of phytoplankton growth to pH in particular, so as to better quantify the likely future biological changes at the regional and global scale.

## Methods

### Model

The coupled atmosphere-ice-ocean carbon cycle model developed by the Commonwealth Scientific Industrial Research Organisation (CSIRO) was used for this study [14]. Details of the model are described elsewhere [2]. Climate change feedbacks were quantified by comparing two separate climate model experiments. The 'control' experiment did not include the warming effects of elevated greenhouse gases in the atmosphere (no radiative forcing) while the 'climate change' experiment explicitly includes the radiative forcing of greenhouse gases in the atmosphere. For both experiments atmospheric CO_2 _levels increased according to observations between 1880 to 1995 then followed IS92a projections until the year 2100 [15]. Differing climate models maintain differing sensitivities to anthropogenic climate forcing. The sensitivity is defined as the global annual temperature change associated with a doubling of atmospheric CO_2_. The sensitivity of the CSIRO Mark II climate model is 4.3°C [16], and is at the higher end of global model sensitivities [15].

## Authors' contributions

BIM initiated the study and RJM developed the carbon cycle model. BIM analysed the model output, provided the main interpretations for the paper and wrote a draft manuscript. RJM provided further interpretations and approved the final version.
